# The Patient’s Denture Assessment (Thai version) is a valid and reliable tool for evaluating the outcome of treatment with complete denture

**DOI:** 10.1186/s12903-021-01405-6

**Published:** 2021-02-08

**Authors:** Sahaprom Namano, Orapin Komin

**Affiliations:** 1 Geriatric Dentistry and Special Patients Care Clinic, Dental Hospital, Faculty of Dentistry, Chulalongkorn University, Bangkok, Thailand; 2 Department of Prosthodontics, Faculty of Dentistry, Chulalongkorn University, 34 Henri Dunant Rd., Pathumwan, Bangkok, 10330 Thailand

**Keywords:** PDA-T, Complete denture, Reliability and validity, Self-assessment, Satisfaction

## Abstract

**Background:**

Complete tooth losses are still being major problems which resulted in lesser quality of life especially for elderly patients. However, there are still lack of questionnaire to evaluate the treatment outcome from the patient’s aspect. The objective of this study is to evaluate the reliability and validity of the Patient’s Denture Assessment-Thai version (PDA-T), then use this questionnaire to assess patient satisfaction with complete denture treatment outcome also investigates the factors involving their satisfaction.

**Methods:**

The subjects comprised 120 edentulous adult patients (49 men/71 women; average age 70 years-old) from the Prosthodontic and the Geriatric Dentistry and Special Patients Care Clinic at the Faculty of Dentistry, Chulalongkorn University during 2019 March‒2020 March. The patients were divided into two groups: the group experienced (Exper) (n = 54) with wearing complete dentures, and the non-experienced (NonExper) group (n = 66). The patients used the validated PDA-T to self-assess their treatment at different times. The Exper group completed the questionnaire at t_0_ (during treatment), t_0.5_ (2‒8-weeks after t_0_), and t_1_ (final follow-up). The NonExper group completed the questionnaire only at t_1_.

**Results:**

In the Exper group, Cronbach’s α and average inter-item correlation was 0.95 (range 0.76‒0.95) and 0.47 (range 0.57‒0.83), respectively. The intraclass correlation coefficients (n = 18, 95% confidence interval) were 0.98 overall. The paired t-test (*p* < 0.05) between t_0_ and t_1_ indicated a significant difference between t_0_ and t_1_ in every PDA-T topic, and the effect size was 1.71. In the NonExper group, the Pearson correlation analysis indicated no significant correlation between the patients' demographics and masticatory function.

**Conclusion:**

The reliability and validity of the PDA-T indicate it is a valuable tool for evaluating complete denture treatment. Treatment success affected the patients' satisfaction but was not associated with the type of doctors, genders, ages, or educational level.

## Background

Although oral health prevention and promotion methods have improved, tooth loss remains a problem at the national level in some countries [1]. According to the 8th National Oral health survey in Thailand, the amount of tooth loss remains high and results in major oral problems. There are many studies on the relationship between tooth loss and oral health related quality of life, specifically in aging people [2, 3]. Moreover, many surveys [4–7] have demonstrated a significant decline in Quality of Life (QoL) when people lose teeth and when denture wearers cannot adapt to their new prosthesis. A study using the Oral Impacts on Daily Performance Index (OIDP) found increased problems from tooth loss and a significantly decreased QoL for the five distal teeth [8]. The prosthesis for an edentulous ridge can be a partial or total denture that restores function and appearance [9]. For edentulous patients, conventional complete denture treatment remains the treatment of choice compared with an implant-retained denture for its simple and inexpensive procedures [10–12].

Many previous studies have used General Oral Health Assessment Index (GOHAI) [13, 14], Oral Health Impact Profile (OHIP) [15], or Oral Impacts on Daily Performance Index (OIDP) [16], to interpret patients’ Oral Health related Quality of Life (OHRQoL) [17, 18]. However, The Patient’s Denture Assessment (PDA) [9], determines QoL based on aspects of wearing a complete denture. The optimum denture treatment outcome requires a careful, systematic evaluation of the existing tissue and oral conditions to accurately fabricate the denture. At every clinical step, dentists and patients (including the patients’ family) share their opinions and evaluate the step results, such as tooth color selection, tooth arrangement try-in or the clinical remount for the occlusion. Furthermore, when using the denture, reevaluation and recall after a period of denture usage is needed. The success of prosthodontic treatment should be evaluated both by dentists and patients aspect [19–21]. Therefore, at the final follow-up visit, the patient’s evaluation of their denture should considered by two-way communication with the dentist. A patient-centered evaluation is an important part of successful denture treatment. Patient satisfaction is usually determined by various factors, including pain, well-fitting, esthetics, retention, stabilization, sense of comfort, and the denture’s chewing ability [9]. A valid and reliable multidimensional self-assessment tool to evaluate patients’ satisfaction and a clinical examination of the denture is needed, so that the dentist can identify the patient-based factors affecting treatment success.

The PDA is an instrument for patient self-assessment. This questionnaire was originally developed in Japanese for edentulous patients with complete dentures at the Tokyo Medical and Dental University, Japan [22]. The PDA allows the patient to self-assess their satisfaction with their complete dentures based on perceptions and feelings [9, 22]. The PDA is used for making a diagnosis, determining the prognosis, and comparing the efficacy of the complete denture (before and after treatment) [9]. Some questionnaires that use many different factors to evaluate the treatment result and measure QoL have not had their reliability and validity determined, however, some methods included several questions concerning the multidimensional evaluation of patient satisfaction [21, 23–25].

The PDA Thai version (PDA-T) was developed using the WHO cross-cultural process. After psychometric (face validity and content validity) testing, an additional question was added to the PDA-T for a total of 23 questions (Table 1) [26]. The purpose of this study was to evaluate the reliability and the validity of the PDA-T, and then use this self-assessment form to evaluate Thai patients’ satisfaction toward the complete denture outcome and also investigates the factors involving their satisfaction which included: type of doctor (undergraduate/postgraduate dental student), ages, genders, and highest education. The hypotheses in this study were that the PDA-T is a valid and reliable tool for evaluating the outcome of the complete denture treatment.

**Table 1:** Question items of PDA-T

**Question 1–4 The use of removable dentures**	
1. Do you have pain while wearing dentures?	
2. Can you easily swallow food or water?	
3. Can you enjoy your meal?	
4. Do you feel jaw discomforting?	
**Questions 5–8 Lower denture functioning**	
5. Are food trapped under the denture base while eating?	
6. Are the dentures properly fitted or not?	
7. Does the lower denture attach smoothly to the gums or not?	
8. Do you feel that the lower denture is in harmony with other parts of the mouth?	
**Question 9–12 Upper denture functioning**	
9. Are food trapped under the denture base while eating?	
10. Does the upper denture attach smoothly to the gums or not?	
11. Does the upper denture move loose while in use?	
12. Do you feel that the upper denture is in harmony with other parts of the mouth?	
**Question 13–15: Dentures expectation**	
13. Do you think your new dentures will meet your expectations?	
14. Do you think there will are any problems with the new dentures?	
15. Do you think that the dentist will create a proper denture for you?	
**Question 16–19 Function of the denture in Beauty and speaking aspect**	
16. Are you worried about the eyes of others who look at you?	
17. Do you feel difficult to speak?	
18. Do you feel concerned about the feature and shape of the area around the lips?	
19. Does the denture clicking sound while chewing?	
**Question 20–23. The importance of using the denture**	
20. Do you think that the denture is considered as part of the body?	
21. Do you think that the denture is significant for you?	
22. How difficult do you think of the denture care of the denture you are using?	
23. Are you comfortable when wearing these dentures?	

## Methods

The patients in this cross-sectional study were randomly selected from the undergraduate clinic (UG), postgraduate Prosthodontic Clinic, and postgraduate Geriatric Dentistry and Special Care Clinic (PG), Dental Hospital, Faculty of Dentistry, Chulalongkorn University, Bangkok, Thailand during 2019 March‒2020 March. The participants were edentulous patients which divided into two group. The first group was those who had received a complete denture before referred as “experienced group” (Exper), and the second group had never received a complete denture referred as “non-experienced group” (NonExper). From G*Power calculation, suggested that at least 46 patients were necessary to find a significant effect: effect size at 0.50, α < 0.50, and 95% power. The inclusion criteria were that the participants could read and respond in Thai and were without any signs of dementia or any mental disabilities. While the exclusion criteria were that the patient cannot respond consciously or understandably and were not involved as a faculty’s patients. The study protocol was approved by the Human Research Ethics Committee at the Faculty of Dentistry, Chulalongkorn University (HREC-DCU 2019–004). Consent forms were signed prior to enrollment to the study.

The patients’ demographic information and chief complaint were collected. The patients’ satisfaction with their complete denture was measured using the PDA-T. In the Exper group, the participants completed the questionnaire two times: during treatment (t_0_), and after the final recheck (t_1_). Eighteen patients were randomly selected from the Exper group to determine test–retest reliability. This was done at any treatment step prior to the final follow-up (t_1_), and the PDA-T was repeated 2–8 weeks (t_0.5_). In the NonExper group, the patient complete the questionnaire only once at t_1_, the final recheck. The data were collected and evaluated by one investigator. Each of the questionnaires’ 6 topics had their own subtopics, comprising groups of items in that particular topic [26].

**Fig. 1:**
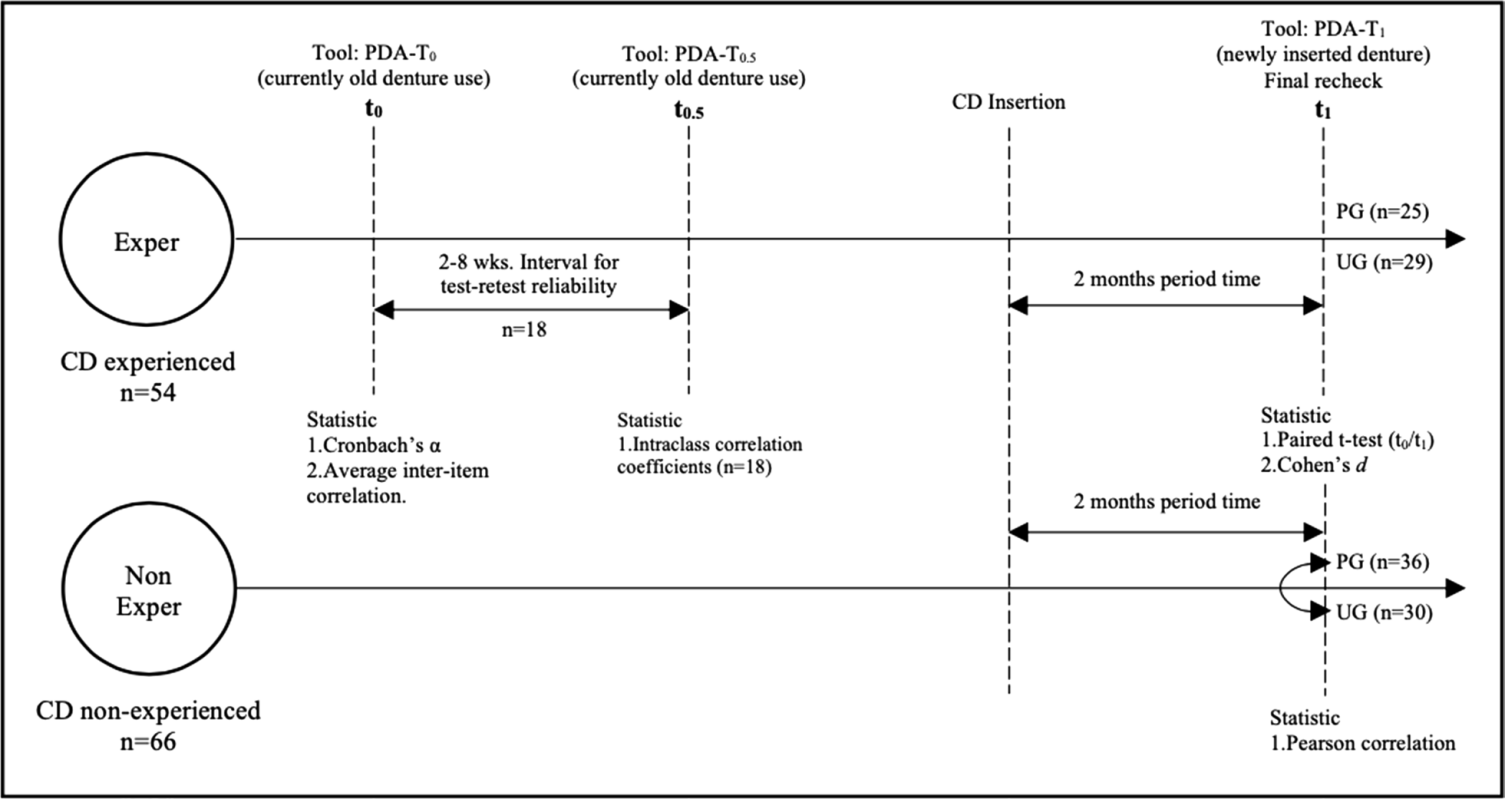
Study flow schematic

The patients were requested to mark their answer on the visual analog scale (VAS), where the right-end was the most positive (100), and the left-end was the most negative (0). The diagram for the study flow schematic was presented in Fig. 1. The Dental students treated the patients under the supervision of the faculty clinician from the first visit to the last visit. Thus, the treatment steps were standardized between cases.

*Exper group*: Internal reliability was determined using the Cronbach’s α coefficient and the average relationship inter-item correlation tests. The ideal range of the average inter-item correlation is 0.15‒0.50 [27, 28]; however, for clinically useful, the range should be between 0.30 and 0.80 [29]. The external reliability in this study, using the test–retest reliability index, was assessed by determining the intraclass correlation coefficient (ICC) and 95% confidence interval of the test–retest difference for 18 patients. The significant range of clinically acceptable agreement ≥ 0.75 [30]. Discriminant validity means the index can accurately distinguish the characteristics measured by other indicators with different theoretical structures [31]. To compare the PDA-T scores between t_0_‒t_1_ in the Exper group, the score was calculated by summing the VAS scores of the question items corresponding to each subtopic. We assessed the differences in PDA-T scores between t_0_ and t_1_ using the paired t-test. Furthermore, the summary score of all the question items was divided by the best possible score (it was 100 score per question). An increased value at t_1_ compared with t_0_ indicated a better score. The effect size compares the efficacy of different treatments by quantifying the size of the difference between treatments. The effect size in the Exper group was determined using Cohen’s *d*. Cohen’s *d* classifies effect sizes as small (*d* = 0.2), medium (*d* = 0.5), large (*d* = 0.8), and very large (*d* ≥ 1.3) [32].

*NonExper group*: The concurrent validity in this study using factors related between the undergraduate (UG) and postgraduate (PG) dental students, age, genders, and highest education with PDA-T score was tested by Pearson’s correlation, using the linear relationship criteria for the result (r^+^: direct linear relationship, r^−^: inverse linear relationship, r^0^: non-linear relationship) [33] with a significance level of 5%.

### Statistical analysis

G-Power version 3.1 software (Erdfelder et al., 1996) was used for NonExper’s sample size analyses. SPSS version 20.0 (SPSS Bangkok, Thailand) was used for all statistical analyses. The sample size and the statistic evaluation were shown in Table 2.

**Table 2:** The sample size and the statistic evaluation for each investigation

Group	Type of investigation	Statistic	N (male/female)	Mean age (years)	UG/PG (n)
Exper	Internal consistency	Cronbach’s α	54 (20/34)	70.3	29/25
		Average inter-item correlation			
	Test–retest reliability	Intraclass correlation coefficients (ICCs)	18 (11/7)	74.5	13/5
	Discriminant validity	Paired t-test	54 (20/34)	70.3	29/25
	Ability to detect change	Cohen’s *d*	54 (20/34)	70.3	29/25
NonExper	Concurrent validity	Pearson correlation	66 (29/37)	69.5	30/36

*Exper group*: The internal consistency of the PDA-T was assessed using Cronbach’s α and average inter-item correlation. The test–retest reliability was assessed with the intraclass correlation coefficients (ICCs) with a 95% confidence interval in 18 random patients (t_0_, t_0.5_). Values of *p* < 0.05 were considered significant for the paired t-test. The ability to detect change was determined based on effect size using Cohen’s *d* criteria.

*NonExper group*: Concurrent validity was determined using Pearson correlation at a significance level of 5%.

## Results

There were 120 patients (49 men, 71 women) in this study. The demographics of the patients (49 men, 71 women; average age 70 years) are presented in Table 3. Of the 120 patients, 54 patients had received complete dentures at least once before and were placed in the Exper group. The remaining 66 patients who had never worn complete dentures before were assigned to the NonExper. We determined the mean and SD in each topic of the PDA-T_0_ and PDA-T_1_ from Table 4. These results revealed a significant increase from t_0_ to t_1_ in all subtopics in the Exper group. However, the Lower denture topic questions had the lowest.

**Table 3:** The denture wearing history of the participants (N = 120)

Characteristics	Group			
	A (n = 54)		B (n = 66)	
	n	%	n	%
Period of edentulousness				
< 1 year	0	0	60	90.9
1 year ≤ 5 years	10	18.5	6	9.1
5 years ≤ 10 years	22	40.7	0	0
≥ 10 years	22	40.7	0	0
Number of previous complete dentures, piece(s)				
Never	N/A	N/A	66	100
1–3	45	83.3	N/A	N/A
4–6	9	16.6	N/A	N/A
Denture problems for requiring a new one				
Ill-fitting complete dentures	35	64.8	N/A	N/A
Malfunction while chewing	18	33.3	61	92.4
Broken/lost previous complete denture	28	51.9	N/A	N/A
Esthetic concern	2	3.7	12	18.2
Extracted natural teeth	N/A	N/A	66	100
Responsible treatment clinic				
Undergraduate	29	53.7	30	45.5
Postgraduate	25	46.3	36	54.5
Highest educational level				
Non-educated	9	16.7	8	12.1
Primary-secondary school	34	63.0	42	63.7
Diplomas	5	9.3	4	6.1
Bachelor’s degree	5	9.3	9	13.6
Master’s degree	1	1.9	3	4.5

**Table 4:** Mean values and standard deviations (SDs) for t_0_ and t_1_ patient’s denture assessment (PDA) scores in Exper and NonExper group (total n = 120)

Topics	Subtopics	Mean ± SD		
		Exper (n = 54)		NonExper(n = 66)
		t_0_	t_1_	t_1_
Function	Q1	68 ± 34	95 ± 10	97 ± 12
	Q2	76 ± 26	95 ± 8	97 ± 10
	Q3	68 ± 30	93 ± 13	93 ± 19
	Q4	79 ± 27	98 ± 5	97 ± 11
Lower denture	Q5	32 ± 28	69 ± 20	72 ± 23
	Q6	33 ± 33	72 ± 20	84 ± 18
	Q7	38 ± 34	75 ± 21	84 ± 18
	Q8	49 ± 35	79 ± 20	88 ± 14
Upper denture	Q9	62 ± 32	96 ± 10	91 ± 20
	Q10	64 ± 32	98 ± 6	96 ± 14
	Q11	57 ± 34	98 ± 5	95 ± 17
	Q12	63 ± 32	98 ± 4	98 ± 6
Expectation	Q13	81 ± 28	98 ± 6	96 ± 15
	Q14	61 ± 39	98 ± 6	95 ± 10
	Q15	84 ± 25	99 ± 4	95 ± 17
Beauty and speech	Q16	78 ± 30	98 ± 5	95 ± 17
	Q17	80 ± 30	99 ± 3	94 ± 16
	Q18	83 ± 24	99 ± 3	97 ± 11
	Q19	78 ± 29	99 ± 3	94 ± 21
Importance	Q20	79 ± 30	99 ± 2	97 ± 13
	Q21	81 ± 29	100 ± 1	98 ± 13
	Q22	79 ± 30	99 ± 4	97 ± 8
	Q23	65 ± 36	99 ± 4	97 ± 7

score compared with the other topics at both evaluation time points, while the least difference in scores between topics was in the Beauty and speech and Importance topics, as well as in Q13 and Q15 in the Expectation topic. At t_1_ in the NonExper group, there were no significant differences between subtopic scores. However, the Lower denture topic had the lowest score (under 90) in every subtopic.

### PDA-T reliability and validity

#### Exper group

The internal consistency using Cronbach’s α and average inter-item correlation was 0.95 (range 0.76‒0.95) and 0.47 (range 0.57‒0.83) (Table 5). The test–retest reliability index assessed by determining the ICCs was 0.98, which indicated clinically significant reproducibility. The ICCs of the six subtopics ranged from 0.94 to 0.99 (Table 6). The results of the assessment of discriminant validity are presented in Table 7. The paired t-test demonstrated that the PDA-T_1_ score was significantly higher compared with the PDA-T_0_.

**Table 5:** Cronbach’s α and Average inter-item correlation coefficients assessed by t_0_-PDA (Exper group) scores (N = 54)

Topics	Cronbach’s α	Average inter-item correlation coefficient
Summary score	0.95	0.47
Function	0.90	0.70
Lower denture	0.85	0.60
Upper denture	0.93	0.77
Expectation	0.76	0.57
Beauty and speech	0.95	0.83
Importance	0.87	0.65

**Table 6:** Test–retest reliability assessed by t_0_ and t_0.5_ patient’s denture assessment (PDA), Exper group, scores (N = 18)

Topics	ICC	95% CI
Summary score	0.98	[0.96, 0.99]
Function	0.99	[0.97, 0.99]
Lower denture	0.98	[0.96, 0.99]
Upper denture	0.94	[0.86, 0.98]
Expectation	0.97	[0.92, 0.98]
Beauty and speech	0.96	[0.89, 0.98]
Importance	0.99	[0.97, 0.99]

**Table 7:** Results of the paired t-test for t_0_ and t_1_ patient’s denture assessment (PDA), Exper group scores

Topics		Mean ± SD	*p*
Summary score	t_0_	1539 ± 495	< 0.00
	t_1_	2155 ± 119	
Function	t_0_	292 ± 105	< 0.00
	t_1_	382 ± 32	
Lower denture	t_0_	152 ± 109	< 0.00
	t_1_	295 ± 77	
Upper denture	t_0_	247 ± 119	< 0.00
	t_1_	291 ± 22	
Expectation	t_0_	226 ± 76	< 0.00
	t_1_	295 ± 14	
Beauty and speech	t_0_	319 ± 106	< 0.00
	t_1_	396 ± 11	
Importance	t_0_	304 ± 107	< 0.00
	t_1_	397 ± 10	

score. The mean summary scores increased from 1539 for the PDA-T_0_ score to 2155 for the PDA-T_1_ score. The pooled SD of all the patients was 360, thus, the Cohen’s *d* value of 1.71 indicated a large effect size.

#### NonExper group

From the sample size of 66, the concurrent validity using Pearson correlation between patients’ demographics and summary score (Table 8) revealed no significant association between clinics, ages, genders, and highest education [34].

**Table 8:** Result of Pearson correlation between patient’s information with summary score of t_1_-PDA of the NonExper group

	Factors			
Topics	Types of patient’s doctor level (UG/PG)	Patient’s age between UG/PG level	Patient’s genders between UG/PG level	Patient’s highest education
Summary score of PDA-T_1_	0.16	0.03	− 0.03	0.05

*p < 0.05

## Discussion

The present study investigated the reliability and the validity of the PDA-T, and then used this self-assessment form to evaluate Thai patients’ satisfaction toward their complete denture experience. The results indicated that the PDA-T can be used to evaluate patient satisfaction with their denture treatment and results.

There is no information about the clinical and oral characteristics of the participants. However, we focused to collect the data from chair-side check and interviewing, randomly. The study participants in each group had problems based on missing teeth, ill-fitting complete dentures and poor chewing ability in the Exper and NonExper group, respectively. These results indicate that the Exper group focused on how well their complete denture fit. In contrast, in the NonExper group, the primary concern was to gain chewing ability according to Table 3. Therefore, Exper group are capable of varying the denture satisfaction which may have more concern problems than the NonExper group that more focusing into their chief complaint. Using the PDA-T, not only we can classify the patient’s problem, but also can be used as a guideline to communicate to varied characteristic of the patients. Which also means that using the PDA-T is patient-centered. Furthermore, the data indicated that the Exper group sought treatment due to a poor fitting denture after a period of having their denture (less than five years, 18.5%; more than five years, 81.4%), which most of the Exper patient experienced no more than 3 set of dentures. After the Cronbach’s alpha were applied to the result of the PDA-T score at t_0_, as shown in Table 5, it proved that the score is reliable. Therefore, the minimum years of having a fitting complete denture in this group was five years. The chief complaint in each group indicates that in edentulous patients a well-fitting and functioning denture is more important compared with their psychological and physical needs. The educational level of the participants’ data illustrated that most patients in each group were at the Primary-secondary school educational level. These results suggest patients with a Primary-secondary school education might suffer from tooth loss at an earlier age.

Both groups' treatment, by dental students, procedures were supervised by their respective faculty members, which standardized the procedures between the groups. In this particular, the supervisors’ data are not included in this study, which may differ the quality of treatment result. However, there were no other factors that differentiated the groups in concurrent validity.

The Exper group demonstrated various denture treatment needs before treatment (t_0_). However, the lower denture topic demonstrated the worst satisfaction scores pretreatment. After treatment, the lower denture subtopic average scores were still the lowest score compared with other subtopics in both groups but significantly higher than their old denture, according to Table 4. Which means that experiences are one of the factors that indicate the improvement of overall treatment satisfaction treatment and new dentures. The lower score of the lower denture’s function may indicate the efficacy of the treatment methods, e.g., an error in tooth arrangement or clinical re-mount. To identify the most sensitive step in denture treatment that affects the denture’s functioning requires further studies that include occlusal schemes and ridge height. The results of the present study demonstrated a high degree of reliability and validity. In the present study, the average inter-item correlation and Cronbach's α was used to determine internal consistency. The Cronbach's α summary score of 0.95 (range 0.76‒0.95) indicated similarity between subtopics, which are acceptable for clinical usefulness. Our results were similar to that of another study using the PDA [9]. However, the expectation topic, which comprised 3 subtopics, demonstrated the lowest score (0.76) between topics, which indicated that the number of questions affected the Cronbach's α score [35]. The average inter-item correlation was significantly different between the summary score (0.47) and the beauty and speech subtopic (0.83, demonstrating that the participant’s psychological and physical needs concerns were lower compared with other subtopics. Furthermore, Q16‒Q19 were similar, which may account for the highest score of the average inter-item correlation coefficients in the beauty and speech subtopic.

The ICCs of the test–retest reliability are typically determined with a 2 to 8 weeks interval between tests [9, 36–38]. All of the subtopic ICCs in our study were all close to 1.00, indicating that the PDA-T is reliable.

There was a significant difference in the summary score and the six subtopics scores before and after replacing the old dentures for the assessment of validity. The PDA-T score was significantly higher at t_1_ compared with t_0_ (*p* < 0.05). The greatest improvement was seen in the lower denture topic (approximately two-fold increase), implying the value of lower denture function. The treatment effect might be related to the dentist’s skill in lower denture fabrication. Thus, future studies should include the lower jaw and alveolar ridge anatomical information to better understand the impact of these factors on denture fabrication and treatment results. These results indicate that the PDA-T can detect differences in patients’ self-assessment between previous and new dentures.

The size effect is the amount of change and the indicator illustrates the effectiveness of the treatment. With an effect size of 1.71, this study demonstrated a large significant difference in scores between after-insertion (t_1_) and before-treatment (t_0_), indicating that an edentulous patient’s value functional ability more than other factors. However, the interval between completing the questionnaire might have affected the amount of change detected. If the participants completed the questionnaire longer after completing denture treatment, there might be a larger difference between the negative and positive effects of the denture treatment.

Most of the NonExper patients faced similar problems according to Table 3, and from the improving score in PDA-T_1_ from Table 4 indicated higher satisfaction in all the NonExper patients. Which can also describe that there was no significant difference between the patient demographics (genders, ages, education, and dentist’s skill level) that might affect the quality of the treatment in any category, according to Table 8. There are studies demonstrating that genders affects oral health, suggesting that females might have better oral health compared with males [39–43]. However, our Pearson correlation analysis found no significant correlation between genders and denture function. It may be intuitive to believe that aging is negatively correlated with oral health and their physical condition [2, 17, 44], however, studies have found no relationship between these factors [45–49]. Therefore, our results indicate that we can improve a patient’s oral health while their physical condition declines, such as restoring tooth loss with a denture that leads to a better quality of life. Social and economic status may be another factor affecting the oral related quality of life.

An educational index is a tool that is often used to determine socioeconomic status, especially in the elderly who do not have income from work. Prior studies demonstrated that education level positively correlates with oral hygiene related quality of life [2, 45, 46, 50–52]. However, other studies have not demonstrated a significant association between with educational level patient satisfaction [23, 34]. In contrast, the present study found a weak association between educational level and denture function. These findings suggest that the satisfaction of edentulous patients receiving a complete denture is controlled by their denture’s function, rather than any demographic aspects. Another aspect is whether the dentist ‘s skill level affects the denture-wearing patients’ the quality of life, which should be further explored in future studies. The fact that the treatment given between groups was supervised by Faculty members may explain why the dentist’s skill level did not affect the PDA-T results.

The present study demonstrated the excellent reliability and validity of the PDA-T. The PDA-T would be useful in clinical practice for understanding the patients’ opinion on their denture’s function, which important for the dentist to understand to be able to provide the best denture treatment. However, this study has limitation due to the short time between denture delivery and the final follow-up; therefore, additional studies are needed. For instance, comparing with OIDP or any standard indexes for quality-of-life evaluation.

## Conclusion

Within the limitation of this study, the present study has demonstrated the reliability and validity of the PDA-T. Complete denture wearers considered denture function more important compared with their psychological and physical needs. It is suggested to use the PDA-T to evaluate the patients’ satisfaction with their denture to generate the optimum treatment results. Treatment successfulness affected the patients' satisfaction but was not associated with the type of doctors, genders, ages, or educational level.

## Data Availability

The datasets used and/or analysed during the current study available from the corresponding author on reasonable request.

## Abbreviations

GOHAI: General Oral Health Assessment Index
OHIP: Oral health impact profile
OIDP: Oral Impacts on Daily Performance Index
OHRQoL: Oral Health related Quality of Life
PDA: Patient’s denture assessment
